# Correction: Krbata et al. Effect of Supercritical Bending on the Mechanical & Tribological Properties of Inconel 625 Welded Using the Cold Metal Transfer Method on a 16Mo3 Steel Pipe. *Materials* 2023, *16*, 5014

**DOI:** 10.3390/ma17030658

**Published:** 2024-01-29

**Authors:** Michal Krbata, Robert Ciger, Marcel Kohutiar, Maria Sozańska, Maroš Eckert, Igor Barenyi, Marta Kianicova, Milan Jus, Nad’a Beronská, Bogusław Mendala, Martin Slaný

**Affiliations:** 1 Faculty of Special Technology, Alexander Dubcek University of Trenčín, 911 06 Trenčín, Slovakia; michal.krbata@tnuni.sk (M.K.); robert.ciger@tnuni.sk (R.C.); maros.eckert@tnuni.sk (M.E.); igor.barenyi@tnuni.sk (I.B.); marta.kianicova@tnuni.sk (M.K.); milan.jus@tnuni.sk (M.J.); 2 Faculty of Materials Engineering, Silesian University of Technology, Krasińskiego 8, 40-019 Katowice, Poland; maria.sozanska@polsl.pl (M.S.); boguslaw.mendala@polsl.pl (B.M.); 3 Institute of Materials and Machine Mechanics, SAS, Dúbravská Cesta 9/6319, 845 13 Bratislava, Slovakia; nada.beronska@savba.sk; 4 Faculty of Mechanical Engineering, Brno University of Technology Technická 2896/2, Královo Pole, 616 69 Brno, Czech Republic; slany.m@fme.vutbr.cz

In the original publication [1], the funder Technology Agency of the Czech Republic (TAČR), TK04020189 was not included.

Martin Slaný was not included as an author in the original publication [1], and the additional affiliation 4 is: Faculty of Mechanical Engineering, Brno University of Technology Technická 2896/2, Královo Pole, 616 69 Brno, Czech Republic; slany.m@fme.vutbr.cz. The corrected Author Contributions statement appears here.

Conceptualization, M.K. (Marcel Kohutiar) and M.K. (Michal Krbata); methodology, R.C.; software, M.K. (Michal Krbata) and N.B.; validation, M.S. (Maria Sozańska), R.C. and M.J.; formal analysis, M.K. (Marcel Kohutiar) and B.M.; investigation, M.E.; resources, M.K. (Marta Kianicova) and M.S. (Martin Slaný); data curation, M.K. (Michal Krbata); writing—original draft preparation, M.K. (Marcel Kohutiar), M.K. (Michal Krbata), M.K. (Marta Kianicova) and M.S. (Martin Slaný); writing—review and editing, I.B. and N.B.; visualization, M.E. and M.J.; supervision, I.B.; project administration, B.M. and M.S. (Maria Sozańska); funding acquisition, M.K. (Marta Kianicova) and M.S. (Martin Slaný). All authors have read and agreed to the published version of the manuscript.

The authors state that the scientific conclusions are unaffected. This correction was approved by the Academic Editor. The original publication has also been updated.
